# LncRNA ENST00000563492 promoting the osteogenesis–angiogenesis coupling process in bone mesenchymal stem cells (BMSCs) by functions as a ceRNA for miR-205-5p

**DOI:** 10.1038/s41419-020-2689-4

**Published:** 2020-06-25

**Authors:** Zhengxiao Ouyang, Tingting Tan, Xianghong Zhang, Jia Wan, Yanling Zhou, Guangyao Jiang, Daishui Yang, Tang Liu

**Affiliations:** 1 Department of Orthopedics, The Second Xiangya Hospital, Central South University, 410011 Changsha, Hunan P. R. China; 2 Department of Immunology, Xiangya School of Medicine, Central South University, 88 Xiangya Rd, 410008 Changsha, Hunan P. R. China

**Keywords:** Mechanisms of disease, Trauma

## Abstract

Pain, physical dysfunction, and mental disorders caused by bone nonunion bring great burden to patients. Bone mesenchymal stem cells (BMSCs) isolated from bone nonunion patients with poor proliferation and osteogenic ability are compared with that from normal bone-healing patients. Long noncoding RNAs (lncRNAs) are a class of RNAs that are more than 200 nucleotides in length, lack an open-reading frame encoding a protein, and have little or no protein-coding function, and could regulate gene expression, which is involved in the regulation of important life activities, such as growth, development, aging, and death at epigenetic, transcriptional, and post-transcriptional levels. In this study, we intended to investigate the difference of lncRNA expression between patients with nonunion and normal fracture healing. Our result found that lncRNA ENST00000563492 was downregulated in bone nonunion tissues. LncRNA ENST00000563492 promotes osteogenic differentiation of BMSCs through upregulating the expression of CDH11. On the other hand, LncRNA ENST0000563492 could improve the osteogenesis–angiogenesis coupling process through enhancing the expression of VEGF during osteogenic differentiation of BMSCs. LncRNA ENST00000563492 functions as a ceRNA for miR-205-5p that was targeting CDH11 and VEGF. LncRNA ENST00000563492 could promote the osteogenesis of BMSCs in vivo. Our result indicated that lncRNA ENST00000563492 may be a new target for bone nonunion.

## Introduction

Although most fractures heal after surgery, 5–10% of patients still develop nonunion after treatment. Pain, limb dysfunction, and psychological disorders caused by nonunion cause great pain to patients and families, increasing the financial burden on families and society^1^. The traditional treatment method included that remove the hardened bone, clean the non-connected end tissue, cut the medullary cavity, fixed, or bone graft. However, with the advancement in surgical techniques and treatment concepts, many scholars have found that for hypertrophic nonunion, direct compression and fixation could result in bone healing. For atrophic nonunion, including nonunion with bone defect, even if the fracture end is not exposed, the treatment of bone truncation alone still cures most of the patients. Although most patients could still heal with simple compression or only bone truncation and transfer, but the healing mechanism is still unclear.

To explore the mechanisms of nonunion and healing, many scholars have conducted related research in cell biology and molecular biology. Boyan et al.^2^ first studied the cells isolated from the scar tissue of the nonunion, which were called nonunion cells (NCs)^2^. It was found that the ability of NCs responding to bone morphogenetic protein (BMP) is similar to that of human bone marrow mesenchymal stem cells (BMSCs). NCs also express similar surface antigens of BMSCs, and have multidirectional differentiation potential^3^. Under suitable conditions, NCs can differentiate into osteoblasts, chondrocytes, and adipocytes, and can express related genes and enzymes, but the proliferation ability and osteogenic differentiation ability are worse than normal. However, limited data are currently available on why the proliferative capacity and osteogenic differentiation ablility of NCs are worse than those of BMSCs and why the proliferation and osteogenic differentiation ability of BMSCs in patients with nonunion are different from those in normal human BMSCs^4,5^.

Long noncoding RNAs (lncRNAs) are a class of RNAs that are more than 200 nucleotides in length, lack an open-reading frame encoding a protein, and have little or no protein-coding function, and regulated gene expression that is involved in the modulation of important biological activities such as growth, development, aging, and death at epigenetic, transcriptional, and post-transcriptional levels^6,7^. LncRNAs also play an important role in the development of certain diseases, such as the most widely studied, malignant tumors^6^. An increasing number of studies have shown that lncRNAs play an important role in maintaining the pluripotent state of stem cells^7^. At present, no study has reported the role of lncRNA in the differentiation of NCs, fracture healing, or formation of nonunion. In view of this, we aim to study the difference in lncRNA expression between patients with nonunion and normal fracture healing through the latest high-throughput sequencing technology. We also explore the role of lncRNA in osteogenic differentiation and bone regeneration mediated by BMSCs.

## Methods

### Sample collection of bone nonunion and normal healing

The research project was approved by the Ethics Committee of Central South University. All patients signed an informed consent form. Samples were collected from 10 patients with nonunion and 14 patients with normal fracture healing. See Table 1 for each specimen information. About 3 mm scar tissue of the fracture site in the nonunion group and the normal healing group was collected during the operation. Clinical diagnosis of nonunion is based on FDA definition: the fracture has not healed for at least 6 months and has no tendency to further heal for 3 months; clinical X-ray examination confirms nonunion, and surgery further confirms the formation of a small amount of scar and callus at the end of the fracture, or only a small amount of scar tissue. Exclusion criteria: patients with infections, tumors, autoimmune diseases and other systemic orthopedic-related diseases, bone nonunion caused by pathological fracture, history of hormone use, and smoking.

**Table 1:** Specimen information.

Specimen no.	Gender	Age (years)	Location	Healing status
No. 1	Male	41	Left humerus	Union
No. 2	Male	47	Right humerus	Nonunion
No. 3	Female	47	Left femur	Nonunion
No. 4	Female	27	Left humerus	Union
No. 5	Male	46	Left tibia	Nonunion
No. 6	Male	48	Right tibia	Union
No. 7	Female	58	Left tibia	Nonunion
No. 8	Male	33	Right femur	Nonunion
No. 9	Male	21	Right fibula	Union
No. 10	Male	25	Left femur	Nonunion
No. 11	Female	44	Right humerus	Union
No. 12	Male	45	Right tibia	Union
No. 13	Male	58	Right humerus	Nonunion
No. 14	Male	24	Right femur	Union
No. 15	Male	42	Left tibia	Union
No. 16	Male	44	Left tibia	Union
No. 17	Female	58	Right humerus	Union
No. 18	Female	25	Left tibia	Union
No. 19	Male	26	Left humerus	Union
No. 20	Male	25	Right femur	Union
No. 21	Female	22	Left femur	Union
No. 22	Male	24	Left tibia	Nonunion
No. 23	Male	48	Right femur	Nonunion
No. 24	Male	31	Left femur	Nonunion

### RNA sequencing

Frozen tissue specimens were ground by repeatedly adding liquid nitrogen and 1 ml of TRIzol solvent. The volume of the samples should not have exceeded 10% of TRIzol. Then, the samples were homogenized using a homogenizer. After complete homogenization, RNA was extracted in a 1.5 ml centrifugal tube. After RNA quality inspection, samples were selected and paired. We matched three pairs of specimens in the healing group and the nonunion group by sex, age, and location. Samples from patient no. 1 and no. 2, no. 5 and no. 12, and no. 10 and no. 14 were grouped into three pairs. Sequencing was completed by Shanghai Jingneng Biotechnology Co., Ltd, and the sequencing platform was Illumina HiSeq 2500.

### Isolation and culture of BMSCs

BMSCs were donated by patients who received orthopedic surgery with written informed consent. The study was approved by the institutional ethics committee of Central South University. Bone marrow aspirate (2 ml) from each donor was inoculated in a 10 cm dish with medium (Dulbeccos modified Eagle's medium [Hyclone, USA], 10% fetal bovine serum (FBS) [Gibco, USA], and 100 U/ml penicillin G, and 100 mg/ml streptomycin [Hyclone, USA]) in humidified air at 37 °C. After 3 days of culture, non-adherent cells were removed from the culture supernatant. The BMSCs adhering to the culture dish was cultured in complete medium that was changed every 2 days. When the cultures were confluent, the cells were detached with 0.25% trypsin (Gibco, USA) and then stored or re-seeded. Cells within 2–5 passages were used in the following experiments. Antibodies against the BMSC markers, including anti-CD90, anti-CD29, anti-CD105, and anti-CD44, were purchased from BD Biosciences and used in FACS analysis.

### Lentivirus-mediated overexpression and knockdown

The coding sequence for lncRNA ENST00000563492 was ligated into pLVX-IRES-Puro vector using PCR primers to amplify the cDNA region. The coding sequence for lncRNA ENST00000563492 shRNA was ligated into the pLKO.1-vector using PCR primers to amplify the shRNA. The pLVX-IRES-Puro and pLVX-IRES-Puro-ENST00000563492 or pLKO.1-Vector and pLKO.1-ENST00000563492 constructs were transfected into the viral packaging cell line HEK293T together with pSPAX2 and pMD2G. Viral supernatant was used for infection of BMSCs.

### Fluorescence in situ hybridization

Cells were cultured on the bottom of confocal dishes and the cell density reached 60–70%. The cells were washed with phosphate-buffered saline (PBS) for 5 min and fixed with 4% paraformaldehyde at room temperature for 10 min. The cells were washed with PBS for 5 min, three times in total. One milliliter of precooled perfusate was added to each dish, and the cells were placed at 4 °C for 5 min. After discarding the perfusate, PBS was added to wash the cells for 5 min, three times in total. Each dish was premixed with 200 ml of pre-hybridized liquid, which was sealed at 37 °C for 30 min, and hybridized liquid preheated at 37 °C; the prehybridization solution was discarded, and a suitable amount of probe hybridization solution containing probe was added. The hybridization solution was sheltered from light and overnight at 37 °C. The cells were washed three times with hybridized lotion I at 42 °C, 5 min each time, then one time with hybridized lotion II and hybridized lotion III, respectively. At last, the cells were washed with PBS at room temperature. DAPI was stained for 10 min, washed with PBS three times, 5 min each time, and observed with confocal microscopy.

### Quantitative reverse transcriptase PCR (QRT-PCR) analysis

Total RNA was extracted using TRIzol reagent, and cDNA was obtained by reverse transcription using PrimeScript RT Master Mix cDNA Synthesis Kit, and qRT-PCR was detected using SYBR Green PCR kit. The qRT-PCR reaction conditions were set as follows: denaturation at 95 °C for 30 s, 50 cycles at 95 °C for 10 s, and 60 °C for 30 s. All measurements were calculated using the 2^−ΔΔCT^ method with GAPDH as an endogenous control.

### RNA pull down

Cells were harvested by trypsinization and resuspended in PBS and freshly prepared with nuclear isolation buffer (2 ml) and water (6 ml). The suspension was on ice for 20 min (with frequent mixing). The nuclei were pelleted by centrifugation at 2500*g* for 15 min. The nuclear pellet was resuspended in freshly prepared RIP buffer (1 ml). The resuspended nuclei were split into two fractions of 500 ml each (for mock and IP). Chromatin was mechanically sheared using a Dounce homogenizer with 15–20 strokes. The nuclear membrane and debris were pelleted by centrifugation at 13,000 r.p.m. for 10 min. The antibody against MS2b-GPF or Ago2 (10 µg) was added to the supernatant (10 mg) and incubated for 2 h (to overnight) at 4 °C with gentle rotation. Protein A/G beads (40 µl) were added and incubated for 1 h at 4 °C with gentle rotation. The beads were pelleted at 2500 r.p.m. for 30 s, the supernatant was removed, and the beads were resuspended in 500 ml of RIP buffer. The process was repeated for a total of three RIP washes, followed by one wash in PBS. Coprecipitated RNA was isolated by resuspending beads in TRIzol RNA extraction reagent.

### Biotin-coupled miRNA capture

The biotin-coupled miRNA pull-down assay was performed using previously reported method^8^. Briefly, the 3′-end biotinylated miR-205-5p mimic or control biotin-miRNA was transfected with BMSCs at a final concentration of 200 nmol/l for 24 h. The biotin-coupled miRNA complex was pulled down by incubating the cell lysate with streptavidin-coated magnetic beads. The abundance of lncRNA and mRNA in bound fractions was evaluated by qRT-PCR analysis.

### Western blot analysis

Total protein was extracted by RIPA buffer, and protein concentration was detected using a BCA protein quantification kit. A 30 μg protein sample was used for 10% SDS-PAGE electrophoresis. After electrophoresis, the protein was transferred to a PVDF membrane, and the membrane was blocked with 5% BSA. Primary antibody was added to the membrane and incubated overnight, and then incubated with an HRP-labeled secondary antibody, and ECL development was performed after the incubation. The antibodies were as follows: COL1A1 (Abcam, #ab34710)^9^, RUNX2 (Abcam, #ab192256)^10^, OCN (Abcam, #ab13418)^11^, p-SMAD1 (Abcam, #ab73211)^12^, t-SMAD1 (Abcam, #ab33902)^13^, CDH11 (Abcam, #ab151302)^14^, VEGF (Abcam, #ab52917)^15^, beta-catenin (Abcam, #ab32572)^16^, Dicer (Abcam, #ab227518), and GAPDH (Abcam, #ab181602)^17^.

### Osteogenic differentiation assay

The cells were washed twice with PBS, fixed with 4% PFA for 15 min, and then stained with alkaline phosphatase (ALP) or Alizarin Red staining solution for 30 min at 37 °C. After staining, the cells were washed twice with PBS and photographed. For quantitative analysis of ALP activity, cells were digested by trypsin, collected, and manipulated according to the ALP activity quantification kit, and absorbance at 450 nm was examined. For semi-quantitative analysis of Alizarin Red staining, 1 ml of 0.1 N NaOH was added, and the absorbance at 480 nm was detected.

### Human Umbilical Vein Endothelial Cells (HUVEC) scratch test

Cells were seeded at a density of 1 × 10^5^ cells/well into a 12-well culture plate and cultured for 12 h using serum-free medium. Using a pipette tip, the cell layer was scratched; the suspended cells were washed away with a medium, and photographed at 0 and 24 h.

### HUVEC transwell migration analysis

Transwell migration assay was performed using transwell inserts (BD Bioscience, USA) with a filter of 8 μm pore. Approximately 5 × 10^4^ cells in serum-free medium were seeded into the upper chamber of the insert pre-coated with Matrigel, and 700 μl of conditional medium was added to the lower chamber. After 24 h of incubation, the cells were fixed with 75% ethanol and stained with crystal violet. Then, cells on the top surface of the membrane were carefully wiped off, and cells on the lower surface were examined with a microscope. Five random fields were photographed for counting purposes, and the average number of migrated cells was used as a measure of migration capacity.

### HUVEC tube-formation analysis

HUVECs were serum-starved for 16 h and then seeded at a density of 4 × 10^4^ per well on growth factor-depleted Matrigel (BD Biosciences, NSW, Australia) in 48-well plates. Conditional medium was added, and the results were quantified 4 h later. Microscopic fields containing tube-like structures formed in the gel were photographed at low magnification (×10). Five fields per test condition were examined.

### Immunofluorescence analysis

Immunostaining was performed using a standard protocol. After stimulation with BMP-2 for 7 days, hBMSCs were incubated with primary rabbit polyclonal antibody against COL1A1 (Abcam, USA) and mouse polyclonal antibodies against VEGF (Abcam, USA) or mouse polyclonal antibodies against COL1A1 (Abcam, USA) overnight at 4 °C, and then primary antibodies were detected using Cy3- or FITC-conjugated anti-mouse or anti-rabbit IgG secondary antibody (Life Technologies, USA). After the final wash, nuclei were counterstained with Hoechst 3342 (Life Technologies, USA) in PBS for 5 min before imaging. The stained sections were immediately observed by laser confocal microscopy (Zeiss, Germany).

### Ectopic bone-formation analysis

Subcutaneous stem cell implantation was performed as previously described^18,19^. Briefly, BMSCs were infected with different lentiviruses. At 24 h after infection, cells were collected, and approximately 3.0 × 10^6^ cells were mixed with β-TCP ceramic particles (50 mg, Shanghai Bio-lu Biomaterials Co., Ltd, Shanghai, China). This mixture was subcutaneously implanted into the dorsal surface of nude mice. After 6 or 12 weeks, the implants were harvested and fixed in 4% paraformaldehyde, decalcified in an ultrasonic decalcification instrument, and then embedded in paraffin. For histological analyses, sections (5 μm) were stained with hematoxylin and eosin (HE).

### Femoral monocortical defect model analysis

The femoral monocortical defect model was established as previously described^20,21^. Briefly, nude mice were placed under general anesthesia, and then the lateral aspect of the right femur was exposed and pushed aside the overlying soft tissues while carefully preserving the periosteum. A monocortical osseous hole (0.8 mm diameter) was created on the femur lateral surface using a round burr attached to a dental drill. Irrigation with saline was used to remove bone dust and fragments. Approximately 5.0 × 10^4^ hBMSCs after different treatments for 24 h were resuspended in a mixture of medium and Matrigel and then transplanted to the osseous hole. A month later, right femurs were fixed in 4% paraformaldehyde for 24 h at 4 °C. The samples were scanned by high-resolution micro-computed tomography (µCT) (SCANCO, Switzerland) with a spatial resolution of 5 μm. Sagittal image sections of model femurs were used to perform 3D histomorphometric analysis. We defined the regions of interest as (i) the hole between the interrupted cortical bone ends, (ii) injured bone marrow, and (iii) periosteal callus outside the hole. Old bone fragments remaining from the drilling were excluded from the regions of interest. A total of 100 consecutive images were used for 3D reconstruction and analysis, covering most of the injured region and periosteal callus. Structural parameters included BMD and bone volume percentage (BV/TV).

### Statistical analysis

Statistical significance was assessed using two-tailed Student’s *t*-test or analysis of variance (ANOVA). All statistical analyses were performed using SPSS software version 19.0. Statistical significance was considered at *p < 0.05 and **p < 0.01. Data are presented as the mean ± SD.

## Results

### lncRNA ENST00000563492 is downregulated in bone nonunion tissue

**Fig. 1:**
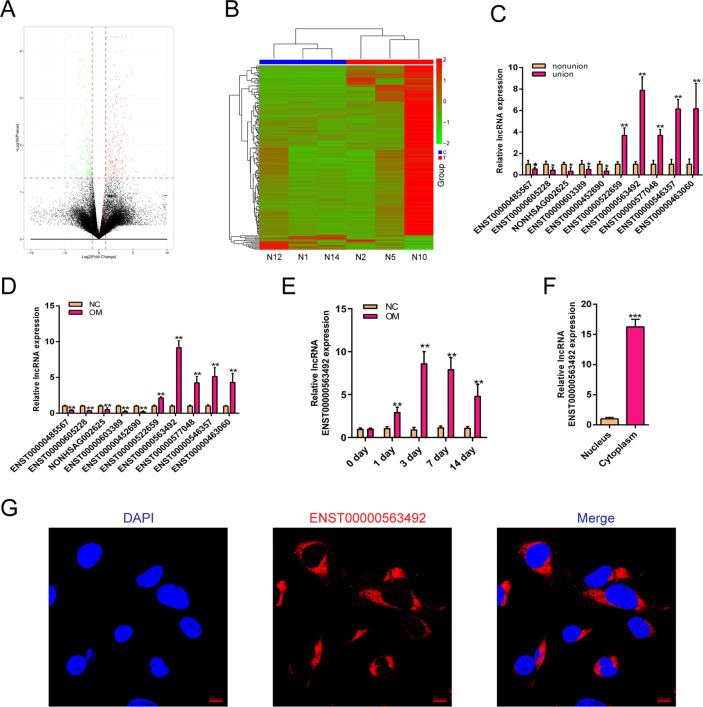
lncRNA ENST00000563492 is downregulated in bone nonunion tissue. **a** Volcano plot of lncRNA expression in nonunion and bone normal fracture-healing tissue. **b** Heatmap of lncRNA expression in nonunion and bone normal fracture-healing tissue. Green, downregulated; red, upregulated. **c** qRT-PCR analysis of lncRNA expression in nonunion and bone normal fracture-healing tissue. BMSCs isolated from patient no. 1–24. **d** qRT-PCR analysis of lncRNA expression in BMSCs on the third day of osteogenesis. BMSCs isolated from patient no. 1, 2, 5, 12, 10, 14, 19, 20, 21, 22, 23, and 24. **e** qRT-PCR analysis of lncRNA ENST00000563942 expression in BMSCs during osteogenesis at 0, 1, 3, 7, and 14 days. BMSCs isolated from patient no. 1, 2, 5, 12, 10, 14, 19, 20, 21, 22, 23, and 24. **f** qRT-PCR analysis of lncRNA ENST00000563942 expression in the nucleus and cytoplasm of BMSCs. NC basal medium, OM osteogenic medium. BMSCs isolated from patient no. 1, 2, 5, 12, 10, 14, 19, 20, 21, 22, 23, and 24. **g** FISH analysis of lncRNA ENST00000563942 in BMSCs. All experiments were performed at least three times. Scale bar = 30 μm. The results are presented as mean ± SD. *p < 0.05, **p < 0.01.

To study the mechanism of lncRNA in the occurrence of nonunion, we first selected 10 patients of nonunion and 14 patients with normal fracture-healing specimens for RNA sequence analysis. The results showed that the expression of lncRNA displayed a large difference in tissues from patients with nonunion and normal fracture healing (Fig. 1a, b). The results of sequencing analysis were verified by qRT-PCR and showed that the expression of lncRNA ENST00000563492 was significantly increased in normal fractured tissue compared with that in nonunion tissue (Fig. 1c). BMSCs were isolated and cultured for osteogenic differentiation, and the expression of lncRNA was detected on the third day of osteogenic differentiation. The results showed that lncRNA ENST00000563492 expression increased most significantly during osteogenic differentiation of BMSCs (Fig. 1d). Further, we examined the expression changes of lncRNA ENST00000563492 during the osteogenic differentiation of BMSCs, and the results showed a significant increase in the early stage of osteogenic differentiation and a decrease in the late stage of 14 days of osteogenesis compared with that at 7 and 3 days (Fig. 1e). To further explore lncRNA ENST00000563492 function, we demonstrated that lncRNA ENST000003492 was mainly localized in the cytoplasm using qRT-PCR and in situ hybridization (Fig. 1f, g).

### LncRNA ENST00000563492 promotes BMSC osteogenesis

**Fig. 2:**
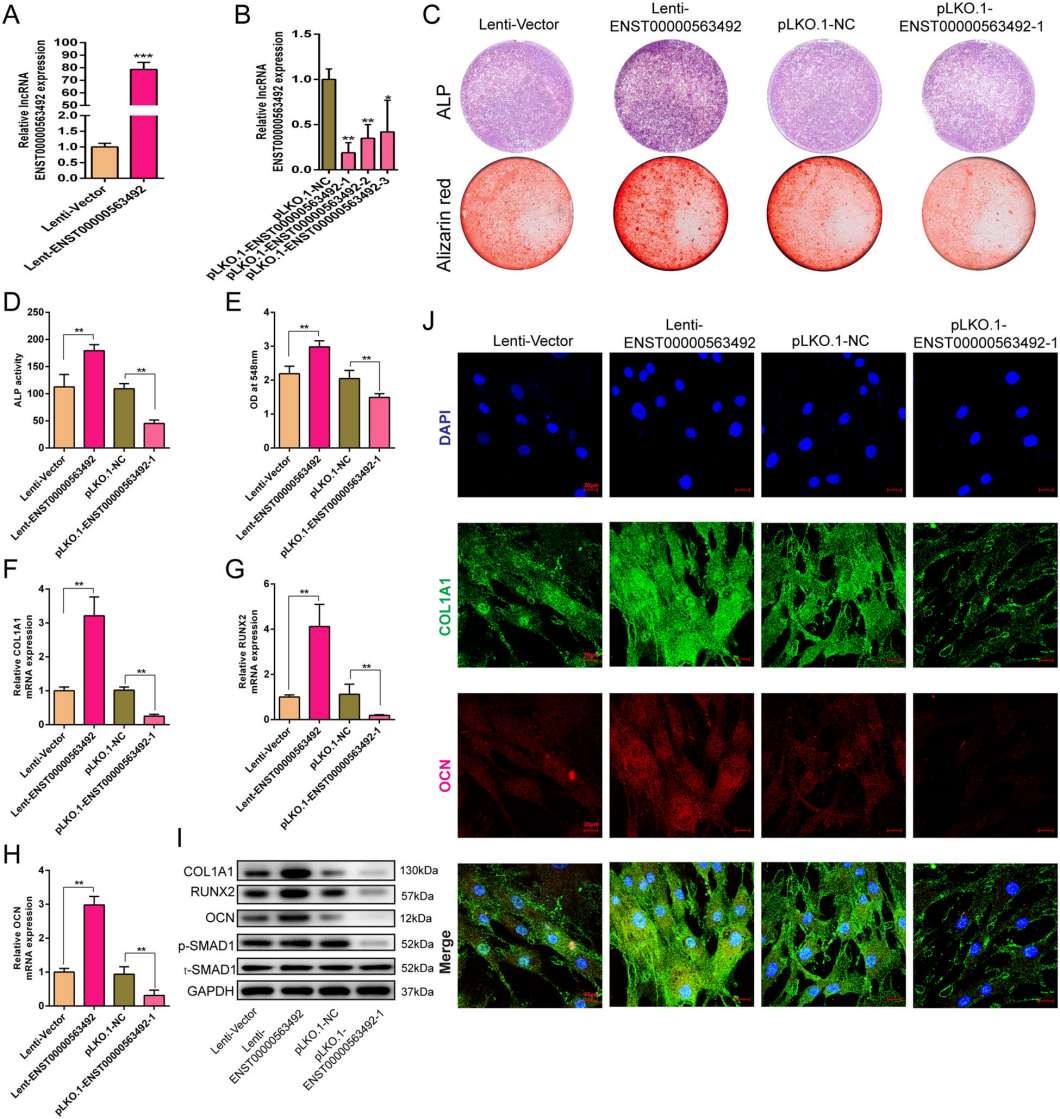
lncRNA ENST00000563492 promotes BMSC osteogenesis. qRT-PCR analysis of the CDH11 mRNA expression in BMSCs after tranfection with Lenti-ENST00000563492 (**a**) and pLKO.1-ENST00000563492 (**b**). **c** ALP activity and Alizarin Red S staining after BMSCs were tranfected with Lenti-ENST00000563492 and pLKO.1-ENST00000563492-1 on the 7th and 14th days. Quantitative analysis of **d** ALP activity and **e** Alizarin Red S staining after BMSCs were tranfected with Lenti-ENST00000563492 and pLKO.1-ENST00000563492-1 on the 7th and 14th day. qRT-PCR analysis of the COL1A1 (**f**), RUNX2 (**g**), and OCN(H) mRNA expression in BMSCs after transfection with Lenti-ENST00000563492 or pLKO.1-ENST00000563492. **i** Western blot analysis of the COL1A1, RUNX2, OCN, p-SMAD1, and t-SMAD1 protein expression after transfection with Lenti-ENST00000563492 or pLKO.1-ENST00000563492. **j** Immunofluorescence analysis of COL1A1 and OCN after BMSCs were tranfected with Lenti-Vector or Lenti-ENST00000563492. NC, basal medium; OM, osteogenic medium. BMSCs isolated from patient no. 1, 2, 5, 12, 10, 14, 19, 20, 21, 22, 23, and 24. All experiments were performed at least three times. Scale bar = 20 μm. The results are presented as mean ± SD. *p < 0.05, **p < 0.01.

We overexpressed and knocked down the expression of lncRNA ENST00000563942 in BMSCs by lentiviruses. qRT-PCR results showed that transfection with lncRNA ENST00000563942 overexpression lentivirus significantly increased the expression of lncRNA ENST00000563942 in BMSCs (Fig. 2a), while that with lncRNA ENST00000563942 knockdown lentivirus significantly decreased the expression of lncRNA ENST00000563942 in BMSCs (Fig. 2b). Overexpression of lncRNA ENST00000563942 enhanced ALP staining and activity, and suppression of lncRNA ENST00000563942 decreased ALP staining and inhibited ALP activity (Fig. 2c, d). Increased expression of lncRNA ENST00000563942 promoted the formation of calcium nodules in BMSCs, and decreased expression of lncRNA ENST00000563942 inhibited the formation of calcium nodules (Fig. 2c, e). LncRNA ENST00000563942 overexpression promotes the expression of COL1A1, RUNX2, and OCN at the mRNA and protein levels, and lncRNA ENST00000563492 knockdown produced the opposite effects (Fig. 2f–i). LncRNA ENST00000563942 could promote phosphorylation of SMAD1 (Fig. 2i). Immunofluorescence assays showed that lncRNA ENST00000563942 could promote the expression of COL1A1 and OCN, and a decrease in the expression of lncRNA ENST000056942 inhibited the expression of COL1A1 and OCN (Fig. 2j).

### LncRNA ENST00000563492 regulates the expression of CDH11 and VEGF

**Fig. 3:**
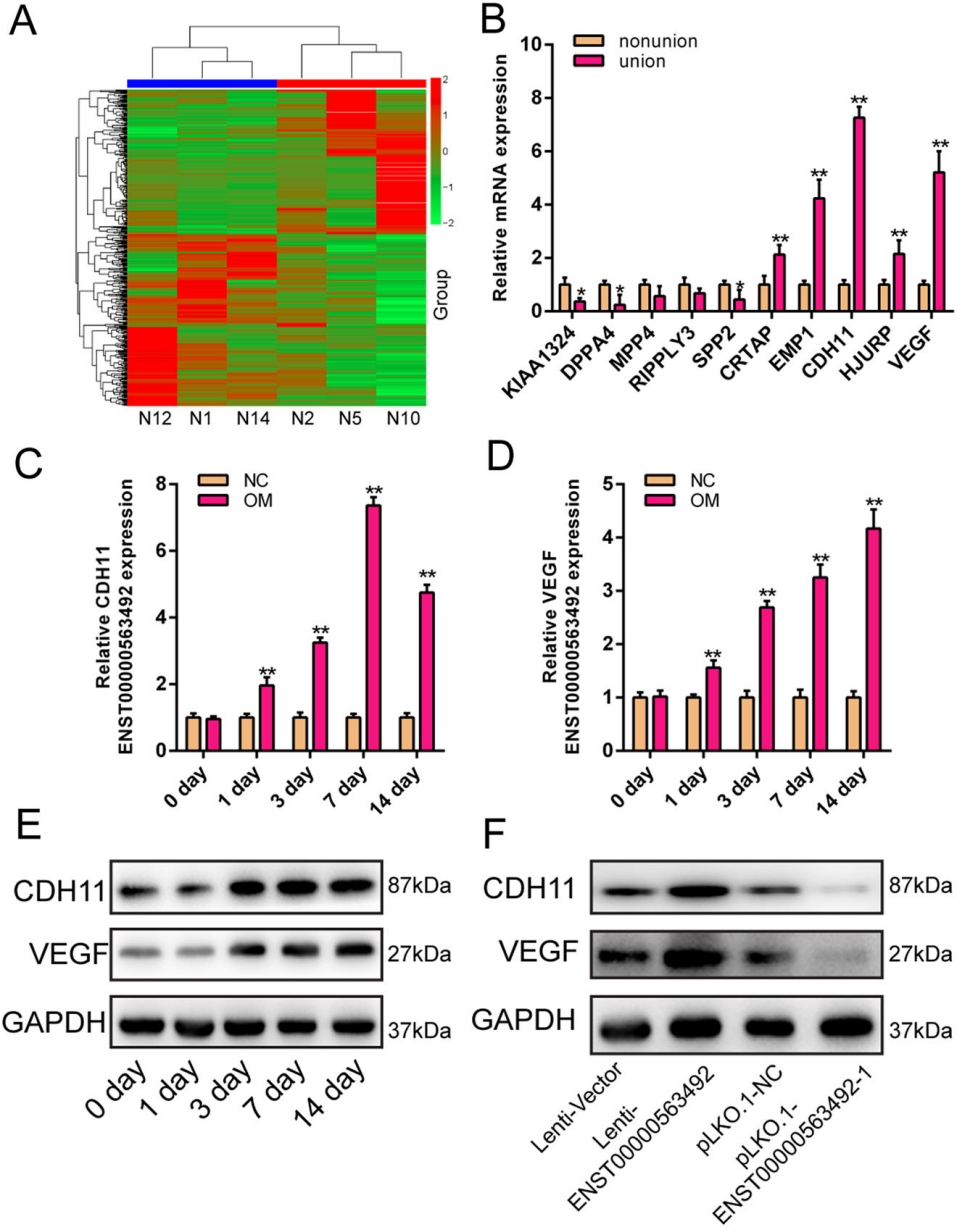
lncRNA ENST00000563492 regulates the expression of CDH11 and VEGF. **a** Heatmap of mRNA expression in nonunion and normal fracture-healing tissues. **b** qRT-PCR analysis of mRNA expression in nonunion and bone normal fracture-healing tissue. qRT-PCR analysis of CDH11 (**c**) and VEGF (**d**) mRNA expression in BMSCs during osteogenesis at 0, 1, 3, 7, and 14 days. **e** Western blot analysis of CDH11 and VEGF mRNA expression in BMSCs during osteogenesis at 0, 1, 3, 7, and 14 days. **f** Western blot analysis of CDH11 and VEGF protein expression in BMSCs transfected with Lenti-ENST00000563492 or pLKO.1-ENST00000563492. NC, basal medium; OM, osteogenic medium. BMSCs isolated from patient no. 1, 2, 5, 12, 10, 14, 19, 20, 21, 22, 23, and 24. All experiments were performed at least three times. The results are presented as mean ± SD. *p < 0.05, **p < 0.01.

To further investigate the mechanism by which lncRNA ENST00000563492 regulates osteogenic differentiation of BMSCs, we analyzed mRNA expression in tissues from patients with nonunion and normal fracture healing. The results showed that there was a large difference in mRNA expression between tissues from patients with nonunion and normal fracture healing (Fig. 3a). We performed qRT-PCR validation on mRNAs with large changes in expression, and the results showed that CDH11 and VEGF were highly expressed in tissues derived from patients with normal fracture healing compared with tissues from patients with nonunion (Fig. 3b). We further examined the increased expression of CDH11 and VEGF during osteogenic differentiation of BMSCs (Fig. 3c–e). Overexpression of lncRNA ENST00000563492 could promote the expression of CDH11 and VEGF, and inhibition of the expression of lncRNA ENST00000563492 could reduce the expression of CDH11 and VEGF (Fig. 3f).

### LncRNA ENST00000563492 promotes BMSC osteogenesis through CDH11/beta-catenin pathway

**Fig. 4:**
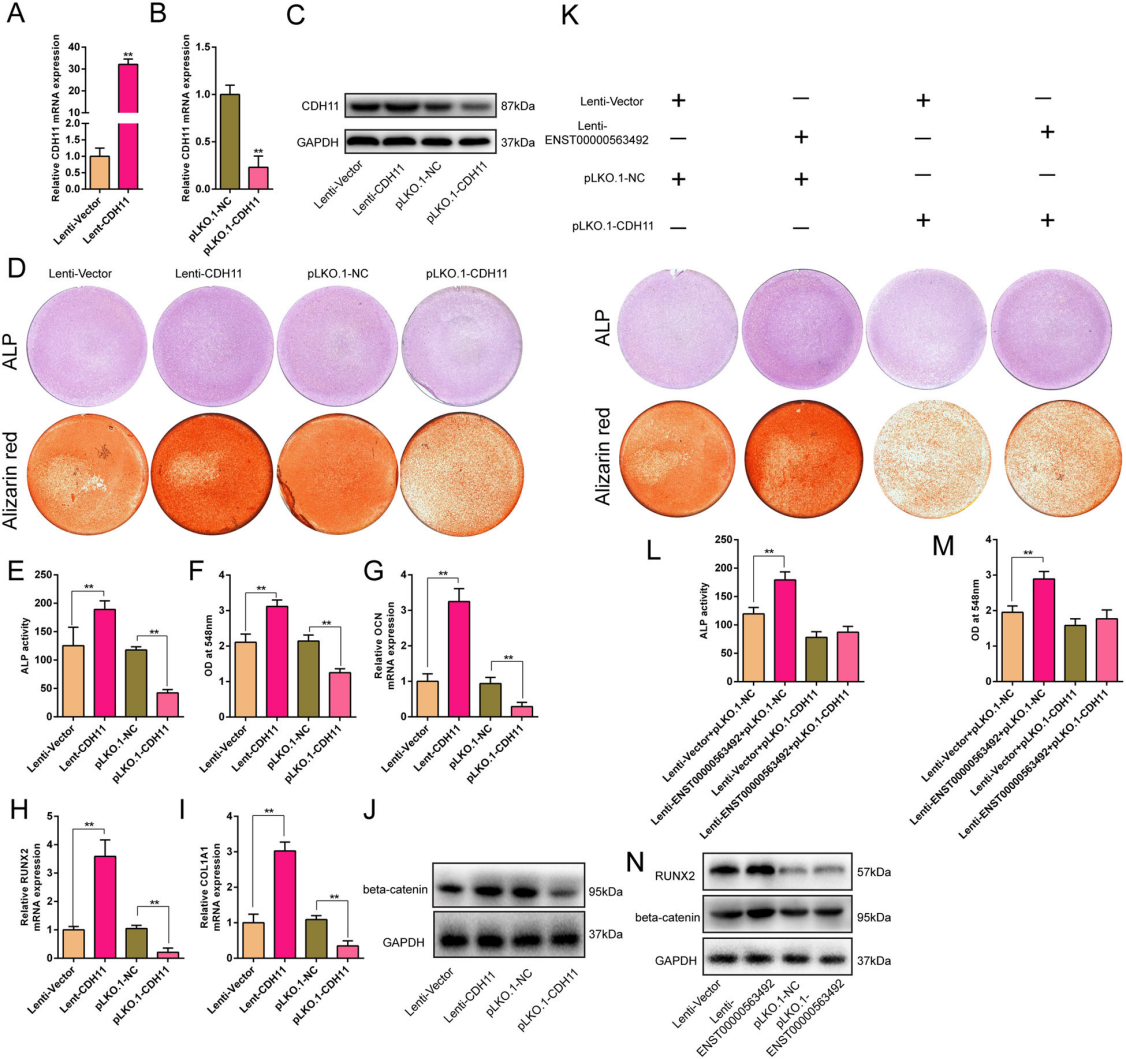
lncRNA ENST00000563492 promotes BMSCs osteogenesis through CDH11/beta-catenin pathway. qRT-PCR analysis of the CDH11 mRNA expression in BMSCs after transfection with Lenti-CDH11 (**a**) or pLKO.1-CDH11 (**b**). **c** Western blot analysis of CDH11 protein expression after transfection with Lenti-CDH11 or pLKO.1-CDH11. **d** ALP activity and Alizarin Red S staining after BMSCs transfected with Lenti-CDH11 or pLKO.1-CDH11 on the 7th and 14th day. Quantitative analysis of **e** ALP activity and **f** Alizarin Red S staining after BMSCs were transfected with Lenti-CDH11 and pLKO.1-CDH11 at the 7th and 14th day. qRT-PCR analysis of OCN (**g**), RUNX2 (**h**), and COL1A1 (**i**) mRNA expression in BMSCs after transfection with Lenti-CDH11 or pLKO.1-CDH11. **j** Western blot analysis of the beta-catenin protein expression after transfection with Lenti-CDH11 or pLKO.1-CDH11. **k** ALP activity and Alizarin Red S staining after BMSCs were transfected with Lenti-Vector and Lenti-ENST00000563492 along with pLKO.1-Vector or pLKO.1-CDH11 on the 7th and 14th day. Quantitative analysis of **l** ALP activity and **m** Alizarin Red S staining analysis after BMSCs. BMSCs were transfected with Lenti-Vector or Lenti-ENST00000563492 along with pLKO.1-Vector or pLKO.1-CDH11 on the 7th and 14th day. **n** Western blot analysis of the RUNX2 and beta-catenin protein expression after transfection with Lenti-Vector and Lenti-ENST00000563492. BMSCs isolated from patient no. 1, 2, 5, 12, 10, 14, 19, 20, 21, 22, 23, and 24. All experiments were performed at least three times. The results are presented as mean±SD. *p < 0.05, **p < 0.01.

To detect the functions of CDH11 in osteogenic differentiation of BMSCs, we overexpressed and knocked down the expression of CDH11 in BMSCs by lentiviruses. qRT-PCR results showed that CDH11 overexpression lentiviruses could significantly increase the expression of CDH11 in BMSCs (Fig. 4a, c), and CDH11 knockdown lentivirus could significantly decrease the expression of CDH11 in BMSCs (Fig. 4b, c). CDH11 overexpression enhanced ALP staining and suppressed ALP activity, and CDH11 knockdown decreased ALP staining and suppressed ALP activity (Fig. 4d, e). Increased expression of CDH11 promoted the formation of calcium nodules in BMSCs, and decreased expression of CDH11 inhibited the formation of calcium nodules (Fig. 4d, f). CDH11 overexpression promoted the expression of OL1A1, RUNX2, and OCN at mRNA levels, and CDH11 knockdown produced the opposite effects (Fig. 4g–i). Furthermore, CDH11 overexpression promoted the expression of beta-catenin, and CDH11 knockdown exerted the opposite effects (Fig. 4j). When the expression of CDH11 was inhibited, the enhancement effect of lncRNA ENST00000563942 on ALP activity and calcium nodules content during the osteogenic differentiation of BMSCs was suppressed (Fig. 4k–o). In contrast, lncRNA ENST00000563492 overexpression promoted the expression of beta-catenin, and lncRNA ENST00000563492 knockdown resulted in the opposite effects (Fig. 4p).

### LncRNA ENST00000563492 promotes angiogenesis of HUVECs

**Fig. 5:**
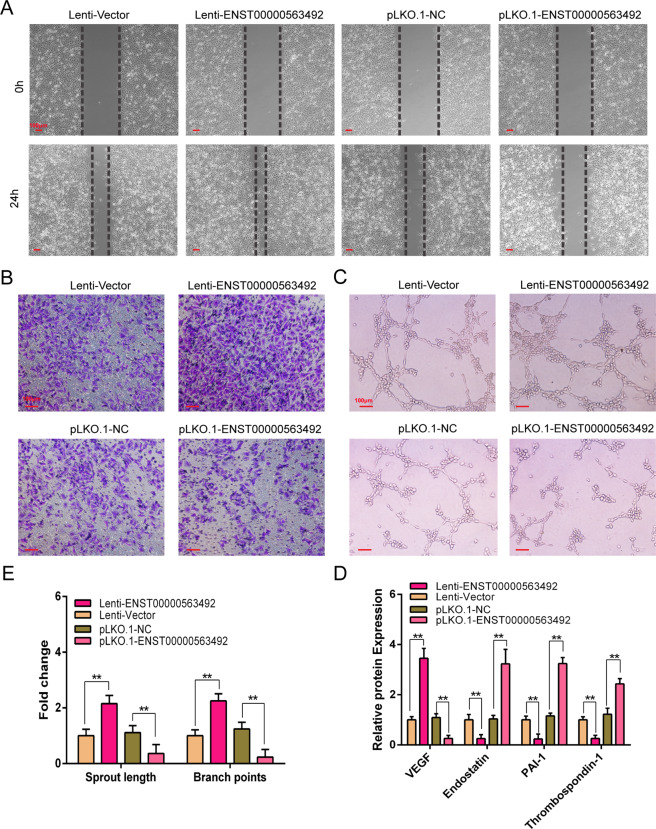
lncRNA ENST00000563492 promotes angiogenesis in HUVECs. **a** Scratch assays were used to detect the migratory ability of HUVECs, and photographs were taken at 0 and 24 h after adding the conditioned medium from BMSCs transfected with Lenti-ENST00000563492, pLKO.1-ENST00000563492, or control plasmid. **b** Transwell assays were used to detect the invasion ability of HUVECs, and photographs were taken at 24 h after adding the conditioned medium from BMSCs transfected with Lenti-ENST00000563492 or pLKO.1-ENST00000563492 or control plasmid in the lower chamber. **c** Tube-formation analysis of angiogenesis in HUVECs and **d** quantitative analysis. **e** ELISA analysis of the secretion of VEGF, endostatin, PAI-1, and thrombospondin-1 in culture medium of BMSCs transfected with Lenti-ENST00000563492, pLKO.1-ENST00000563492, or control plasmid. BMSCs isolated from patient no. 1, 2, 5, 12, 10, 14, 19, 20, 21, 22, 23, and 24. All experiments were performed at least three times. Scale bar = 100 μm. The results are presented as mean ± SD. *p < 0.05, **p < 0.01.

After overexpression and knockdown of lncRNA ENST00000563492 in BMSCs followed by osteogenic differentiation, the culture supernatants from BMSCs were used to culture HUVECs for scratch, transwell, and tube-formation experiments. Scratch experiments showed that culture supernatant from BMSCs with overexpression of lncRNA ENST00000563492 enhanced HUVEC migration, whereas culture supernatant from BMSCs with knockdown of lncRNA ENST00000563492 expression reduced HUVEC migration (Fig. 5a). Transwell experiments showed that culture supernatant from BMSCs with overexpression of lncRNA ENST00000563492 enhanced HUVEC invasion, and culture supernatant from BMSCs with knockdown of lncRNA ENST00000563492 reduced the invasion of HUVECs (Fig. 5b). Angiogenesis experiments showed that culture supernatant from BMSCs with overexpression of lncRNA ENST00000563492 enhanced the angiogenesis of HUVECs, and conversely, culture supernatant from BMSCs with knockdown of lncRNA ENST00000563492 suppressed angiogenesis of HUVEC (Fig. 5c, d). Enzyme-linked immunosorbent assay was used to detect pro-angiogenic and angiogenic factor expression in culture supernatant from cells with overexpression of lncRNA ENST00000563492, and increased expression of VEGF was observed; in contrast, in the culture supernatant from BMSCs with knockdown of lncRNA ENST00000563492 expression, the expression of VEGF was reduced (Fig. 5e).

### LncRNA ENST00000563492 functions as a ceRNA for miR-205-5p to regulate the expression of CDH11 and VEGF

**Fig. 6:**
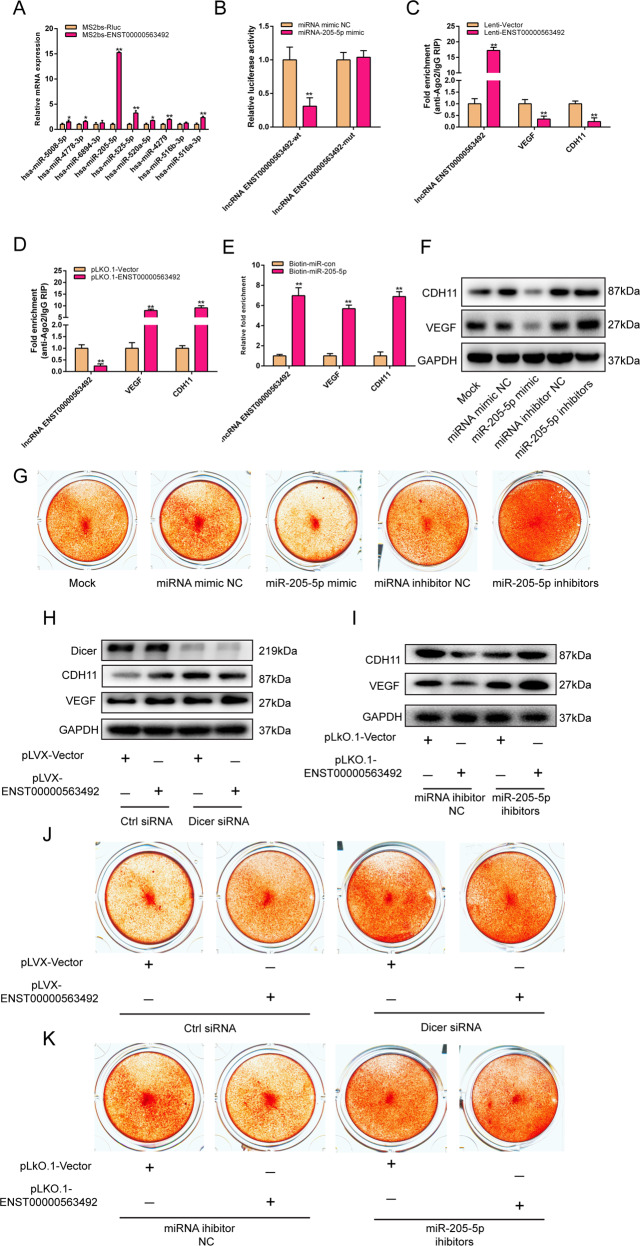
lncRNA ENST00000563492 functions as a ceRNA for miR-205-5p to regulate the expression of CDH11 and VEGF. **a** MS2bs-based RIP assay in BMSCs 48 h after transfection with MS2bp-YFP plasmid along with MS2bs-ENST00000563492 or MS2bs-Rluc (control vectors). **b** Luciferase activity of psiCHECK2-ENST00000563492-wt and psiCHECK2-ENST00000563492-mut upon transfection of NC miRNA mimics or miR-205-5p mimics into HEK293T cells. **c** RIP assay of the enrichment of Ago2 on lncRNA ENST00000563492, CDH11, and VEGF transcripts relative to IgG in BMSCs transfected with Lenti-Vector or Lenti- ENST00000563492. **d** RIP assay of the enrichment of Ago2 on lncRNA ENST00000563492, CDH11, and VEGF transcripts relative to IgG in BMSCs transfected with pLKO.1-Vector or pLKO.1-ENST00000563492. **e** miRNA-RIP assay of the enrichment of lncRNA ENST0000056349, CDH11, and VEGF transcript on miR-205-5p relative to NC miRNA mimics in BMSCs transfected with biotin-miR-con or biotin-miR-205-5p. **f** Western blot analysis of CDH11 and VEGF expression in BMSCs transfected with miR-205-5p mimics, miR-205-5p inhibitors or NC miRNA mimcs and miRNA inhibitors. **g** Alizarin Red S staining analysis of BMSCs transfected with miR-205-5p mimics, miR-205-5p inhibitors or NC miRNA mimics and miRNA inhibitors. **h** Western blot analysis of CDH11 and VEGF expression in BMSCs transfected with Lenti-Vector or Lenti-ENST00000563492 along with Dicer ctrl siRNA or Dicer siRNA. i Western blot analysis of CDH11 and VEGF expression in BMSCs transfected with pLKO.1-Vector or pLKO.1-ENST00000563492 along with NC miRNA inhibitors or miR-205-5p inhibitors. **j** Alizarin Red S staining analysis of BMSCs transfected with Lenti-Vector or Lenti-ENST00000563492 along with Dicer ctrl siRNA or Dicer siRNA. **k** Alizarin Red S staining analysis of BMSCs transfected with pLKO.1-Vector or pLKO.1-ENST00000563492 along with NC miRNA inhibitors or miR-205-5p inhibitors. BMSCs isolated from patient no. 1, 2, 5, 12, 10, 14, 19, 20, 21, 22, 23, and 24. All experiments were performed at least three times. The results are presented as the mean ± SD. *p < 0.05, **p < 0.01.

To further investigate the mechanism by which lncRNA ENST00000563492 regulates CDH11 and VEGF expression, we used bioinformatics analyses to predict miRNAs that bind to lncRNA ENST00000563492. Using MS2bs-based RNA pull-down assay, we found that lncRNA ENST00000563492 can bind to miR-205-5p (Fig. 6a). We mutated the binding site and constructed a luciferase reporte. Luciferase assays also showed that miR-205-5p can bind with lncRNA ENST00000563492 (Fig. 6b). Using bioinformatics analyses, miR-205-5p was found to target the 3′-UTRs of CDH11 and VEGF mRNA. We used the Ago2 RIP assay to find that CDH11 and VEGF competed with lncRNA ENST00000563492 for binding to Ago2 (Fig. 6c, d). On the other hand, by biotin-miRNA-RNA pull-down assay, we found that miR-205-5p can bind with CDH11, VEGF, and lncRNA ENST00000563492 (Fig. 6e). miR-205-5p can significantly reduce CDH11 and VEGF protein levels, and inhibition of miR-205-5p function could promote the expression of CDH11 and VEGF (Fig. 6f). miR-205-5p could significantly reduce the osteogenesis of BMSCs, and inhibition of miR-205-5p function could promote the osteogenesis of BMSCs (Fig. 6g). Suppression of the expression of Dicer inhibited the effect of lncRNA ENST00000563492 on the expression of CDH11 and VEGF (Fig. 6h). When the function of miR-205-5p was suppressed, the effect of lncRNA ENST00000563492 on CDH11 and VEGF expression was also reduced (Fig. 6i). Suppression of the expression of Dicer inhibited the effect of lncRNA ENST00000563492 on the osteogenesis of BMSCs (Fig. 6j). When the function of miR-205-5p was suppressed, the effect of lncRNA ENST00000563492 on the osteogenesis of BMSCs was inhibited (Fig. 6k).

### LncRNA ENST00000563492 improves BMSC osteogenesis and bone regeneration in vivo

**Fig. 7:**
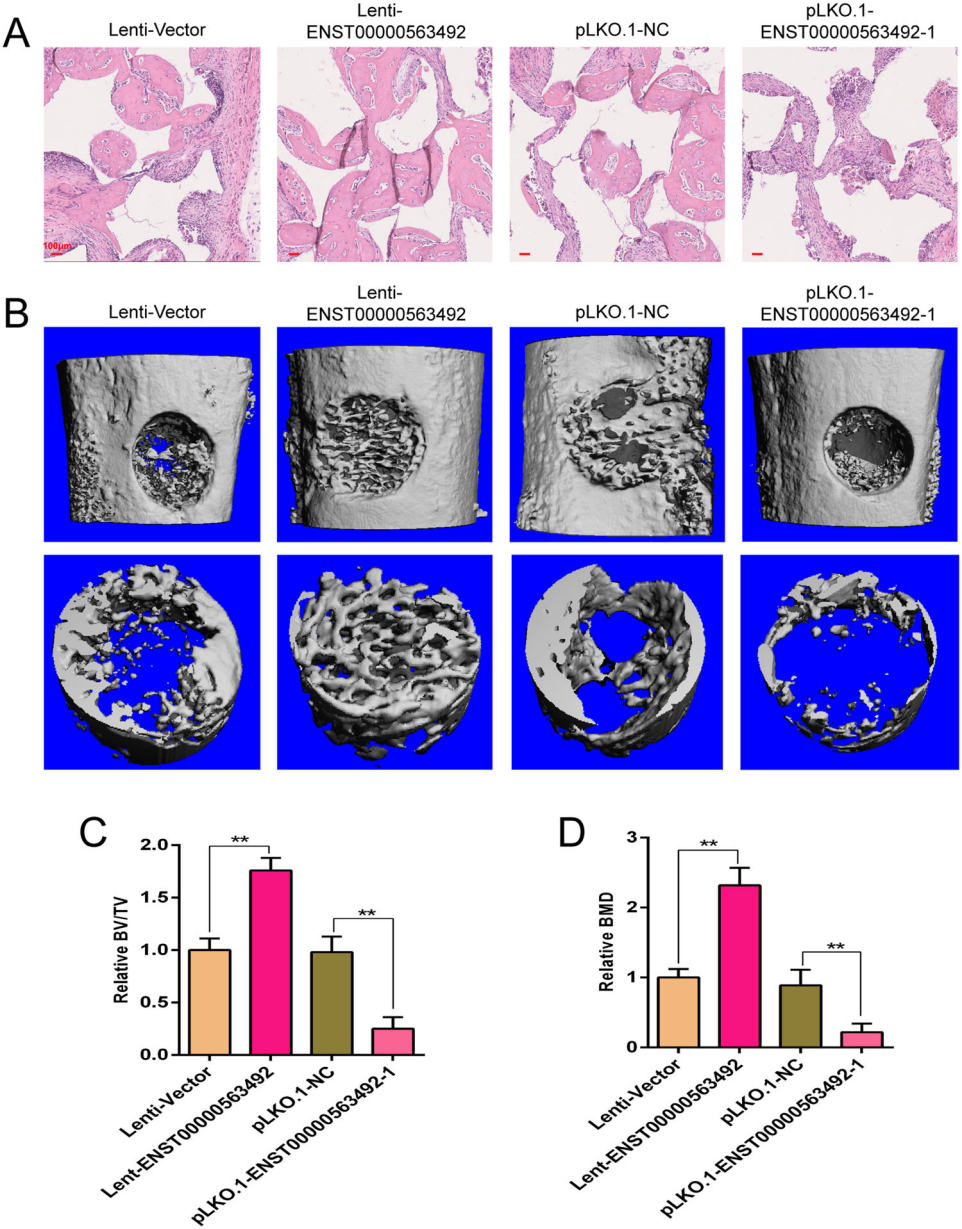
lncRNA ENST00000563492 improves BMSC osteogenesis and bone regeneration in vivo. **a** Representative images of HE staining showing new bone formation (magnification: ×100). **b** Lateral views of a 3D reconstruction of injured femoral (top panel) and mineralized bone formed in the hollowed region (lower panel) by μCT. **c** Relative 3D restructuration parameters BMD of mineralized bone formed in the hollowed region by μCT. **d** Relative 3D restructuration parameters BV/TV of mineralized bone formed in the hole region by μCT. BMSCs isolated from patient no. 1, 2, 5, 12, 10, 14, 19, 20, 21, 22, 23, and 24. *N* = 6. Scale bar = 100 μm. The results are presented as mean ± SD. *p < 0.05, **p < 0.01.

To detect the effect of LncRNA ENST00000563492 on bone regeneration, we used an ectopic osteogenesis model to find that LncRNA ENST00000563492 could significantly promote osteogenic differentiation in BMSCs in vivo and that a reduction in the expression of LncRNA ENST00000563492 could inhibit the osteogenic differentiation of BMSCs in vivo (Fig. 7a). At the same time, lncRNA ENST00000563492 overexpression significantly promoted bone regeneration, and lncRNA ENST00000563492 knockdown inhibited bone regeneration in bone defect model (Fig. 7b–d).

## Discussion

Since lncRNAs are widely involved in epigenetic, transcriptional, and post-transcriptional gene expression, they participate in the biological regulation of growth, development, aging disease, etc., and in recent years, ENCODE has decided to disclose the results of staged research, showing that lncRNA has a far-reaching effect. More extensive than imagined, the mode of action of lncRNAs is complex, gradually bringing lncRNA research to the forefront and a new topic of investigation in the field of life sciences in recent years. The earliest and most important field of lncRNA research is still tumor biology. H19, PCA3, HULC, MALAT1, and HOTAIR are all key lncRNAs^22–24^. In orthopedics, the study of lncRNA is still uncommon. In 2013, Zhu and Xu^25^ found that lncRNA-ANCR inhibits the expression of RUNX2 gene by acting on the EZH2 enhancer. LncRNA-TUG1 can inhibit the proliferation of osteosarcoma cells and promote their apoptosis^26^. LncRNA-H19 upregulated miR-675-3p and miR-675-5p to promote the differentiation and regeneration of skeletal muscle cells^27^. Liu et al.^28^ used gene chip technology and found that the expression of lncRNA-CIR in injured chondrocytes was significantly increased and that this increase promoted the degradation of the extracellular matrix of chondrocytes, aggravating osteoarthritis. In addition, there are few reports on lncRNA in orthopedics. The functions of lncRNAs in bone nonunion had not been reported so far. Through high-throughput sequencing, we have discovered that lncRNA ENST00000563492 is closely related to nonunion and studied its functional mechanism in this study.

Iwakura et al.^3^ found that NCs have similar characteristics to BMSCs, can express similar surface antigens, and have multidirectional differentiation potential. Under suitable conditions, NCs can differentiate into osteoblasts, chondrocytes, and adipocytes, and can express related genes and enzymes, but the proliferation ability and osteogenic differentiation ability are worse than those of normal cells. Some scholars have also studied the difference in BMSCs in patients with nonunion. Hernigou and Beaujean^29^ first studied the proliferative activity of bone marrow cells in nonunion using cell cloning techniques in vitro. Their study found that not only the content of progenitor cells was lower but also the distribution of progenitor cells was more sparse in bone marrow at the site of nonunion than under normal conditions. Researchers from the Seebach C team compared the proliferative activity of BMSCs in patients with multiple lesions, single fractures, and atrophic nonunion, and found that the proliferation of BMSCs was more robust in patients with multiple injuries than in patients with atrophic nonunion^30^. Mathieu et al.^31^ further explored the hypothesis proposed by their predecessors and showed that the proliferative capacity and osteogenic differentiation ability of BMSCs in patients with atrophic nonunion were worse than those of normal BMSCs but that under normal conditions, BMSC function could be restored.

Nonunion is a common orthopedic disease that often occurs in fractures and large bone defects, which limits the patient’s ability to exercise and imposes a heavy financial burden on patients^32,33^. Approximately 5–10% of patients with fracture experience nonunion. The primary principle for the treatment of nonunion is to increase the biological activity of the bone^32^. When a fracture occurs, BMSCs migrate to the bone lesion and differentiate into osteoblasts, secreting a large amount of extracellular matrix to promote fracture healing^34^. BMSC migration and osteogenic differentiation are regulated by a variety of cellular signals^35,36^. Improving the osteogenic differentiation ability of BMSCs is essential for the treatment of nonunion. Our study showed that lncRNA ENST00000563492 expression is significantly elevated during the early stage of osteogenic differentiation and could promote the osteogenic differentiation of BMSCs; thus, ENST00000563492 may be an important molecule determining the fate of BMSCs.

Bone regeneration is accompanied by the ordered production of blood vessels in both space and time^37^. Accordingly, optimization of effective osteogenesis-coupling angiogenesis promoting vascularization is essential for fracture healing and bone nonunion^38^. The release of VEGF from BMSCs during osteogenic differentiation promotes endothelial cell migration and angiogenesis. In this study, we also confirmed that BMSCs release VEGF during osteogenic differentiation to promote HUVEC angiogenesis. In this study, we also confirmed that BMSC releases VEGF during osteogenic differentiation to promote HUVEC angiogenesis. Studies have reported that VEGF plays an important role in nonunion. Overexpression of lncRNA ENST00000563492 in BMSCs significantly promoted the expression of VEGF in BMSC culture supernatant. Therefore, lncRNA ENST00000563492 may play a role in the occurrence of nonunion through two aspects. On the one hand, lncRNA ENST00000563492 directly regulates the osteogenic differentiation of BMSCs, and on the other hand, lncRNA ENST00000563492 indirectly regulates the formation of blood vessels. In vivo, we found that lncRNA ENST00000563492 could promote BMSCs to repair bone defects. Therefore, BMSCs modified by lncRNA ENST00000563492 may be effective seed cells in bone tissue engineering. The use of BMSCs modified by lncRNA ENST00000563492 may be a new method to treat nonunion.

As a tumor suppressor, miR-205-5p can inhibit the growth and invasion of cancer cells and repress angiogenesis induced by cancer cells^39–41^. Similarly, the proliferation and migration of BMSCs are critical for fracture healing. Therefore, the effect of miR-205-5p on the proliferation and migration of BMSCs needs further study. In this study, we found that miR-205-5p could inhibit the expression of CDH11 and VEGF in BMSCs. Further studies showed that miR-205-5p inhibited osteogenesis–angiogenesis coupling. Enhancing bone formation and angiogenesis is key for the treatment of fractures, bone nonunion, delayed healing of fractures, and other related diseases. Bone regeneration occurs in close spatial and temporal association with angiogenesis. Thus, promoting vascularization by optimizing effective osteogenesis–angiogenesis coupling is essential for fracture healing. Therefore, miR-205-5p may influence fracture healing by affecting the proliferation, migration, and angiogenesis of BMSCs. The correlation between miR-205-5p and nonunion needs further study. miR-205-5p may be a new target for the treatment of nonunion.

### Conclusion

In this study we found that lncRNA ENST00000563492 is downregulated in hBMSCs isolated from bone nonunion patients, which promote CDH11 and VEGF expression by downregulating the function of miR-205-5p. Our results explained, at least in part, that the reduced expression of lncRNA ENST00000563492 is an important cause of nonunion, and suggested a new therapeutic strategy for the treatment of bone nonunion.
